# Molecular quantification and differentiation of *Candida* species in biological specimens of patients with liver cirrhosis

**DOI:** 10.1371/journal.pone.0197319

**Published:** 2018-06-13

**Authors:** Sandra Krohn, Katharina Zeller, Stephan Böhm, Antonis Chatzinotas, Hauke Harms, Jan Hartmann, Anett Heidtmann, Adam Herber, Thorsten Kaiser, Maud Treuheit, Albrecht Hoffmeister, Thomas Berg, Cornelius Engelmann

**Affiliations:** 1 University Hospital Leipzig, Section of Hepatology, Clinic of Gastroenterology and Rheumatology, University Hospital Leipzig, Leipzig, Germany; 2 Helmholtz Centre for Environmental Research—UFZ, Department of Environmental Microbiology, Leipzig, Germany; 3 Ludwig Maximilians-University, Max von Pettenkofer-Institute for Hygiene and Clinical Microbiology, Munich, Germany; 4 Department of Gastroenterology and Hepatology, Hospital and Outpatient Clinic for Internal Medicine A, Ernst Moritz Arndt University of Greifswald, Greifswald, Germany; 5 Institute for Laboratory Medicine, Clinical Chemistry and Molecular Diagnostics, University Hospital Leipzig, Leipzig, Germany; 6 Interdisciplinary Endoscopy Unit, Clinic of Gastroenterology and Rheumatology, University Hospital Leipzig, Leipzig, Germany; 7 University College London, Institute for Liver and Digestive Health, Royal Free Campus, London, United Kingdom; Medizinische Fakultat der RWTH Aachen, GERMANY

## Abstract

Patients with liver cirrhosis are susceptible to fungal infections. Due to low sensitivity of culture-based methods, we applied a real-time PCR assay targeting the 18S rRNA gene in combination with direct sequencing and terminal-restriction fragment length polymorphism (T-RFLP) in order to establish a novel tool to detect fungal DNA and to quantify and differentiate *Candida* DNA, also in polyfungal specimens. In total, 281 samples (blood n = 135, ascites n = 92, duodenal fluid n = 54) from 135 patients with liver cirrhosis and 52 samples (blood n = 26, duodenal fluid n = 26) from 26 control patients were collected prospectively. *Candida* DNA was quantified in all samples. Standard microbiological culture was performed for comparison. Blood and ascites samples, irrespective of the patient cohort, showed a method-independent low fungal detection rate of approximately 1%, and the Candida DNA content level did not exceed 3.0x10^1^ copies ml^-1^ in any sample. In contrast, in duodenal fluid of patients with liver cirrhosis high fungal detection rates were discovered by using both PCR- and culture-based techniques (81.5% vs. 66.7%; p = 0.123) and the median level of *Candida* DNA was 3.8x10^5^ copies ml^-1^ (2.3x10^2^-6.3x10^9^). In cirrhosis and controls, fungal positive culture results were confirmed by PCR in 96% and an additional amount of 44% of culture negative duodenal samples were PCR positive. Using T-RFLP analysis in duodenal samples, overall 85% of results from microbial culture were confirmed and in 75% of culture-negative but PCR-positive samples additional *Candida* species could be identified. In conclusion, PCR-based methods and subsequent differentiation of *Candida* DNA might offer a quick approach to identifying *Candida* species without prior cultivation.

## Introduction

The incidence of fungal infections is increasing in patients with end-stage liver diseases and contributes to high fatality rates [1–3]. Fungal infections most often evolve endogenously as fungal pathogens grow uncontrolled in the gastrointestinal tract and migrate into the systemic circulation. This so-called intestinal translocation is promoted and maintained by a cirrhosis-associated impaired intestinal barrier function and a dysfunctional immune system [4–6]. When fungal infections develop, it is of high importance that pathogens are identified rapidly and reliably in order to enable early and adequate antifungal treatment. However, low sensitivity of culture-based standard diagnostic procedures and serological *Candida* antigen assays, as well as time-consuming microbial identification methods [1, 7, 8] have resulted in delayed treatment initiation, which contributes to the high numbers of deaths related to cirrhosis.

Culture-independent PCR-based approaches to rapidly detecting DNA from fungal species have already been developed and evaluated [9, 10]. The amplification of variable sequences of the 18S ribosomal RNA (rRNA) gene or its non-coding internal transcribed spacer region has shown high sensitivities [11, 12]. However, the PCR-based methodology has certain limitations. First, false positive results can be created by not using ultra-clean reagents whereas endogenous PCR inhibitors may cause false-negative results [13]. Second, multispecies identification in fungal PCR followed by subsequent sequencing is difficult due to the lack of a web-based tool for the differentiation of polyfungal amplicons, a platform which already exists for mixed bacterial samples [14]. The latter problem can be addressed using PCR-RFLP techniques, which at least allow *Candida* species to be identified. However, time-consuming prior cultivation of fungal microorganisms is needed and only a small panel of *Candida* species can be distinguished [15–17].

We developed a novel PCR-based method to identify and differentiate fungal DNA. Ultra-clean reagents for DNA isolation and enrichment of fungal DNA were used to overcome the problem of false-positive PCR results. The use of panfungal primers and *Candida*-specific hybridization probes in one PCR setting, enables us to qualitatively detect a broad spectrum of fungal genera and simultaneously quantify DNA from *Candida* species that represent the most prominent fungal pathogens detected in human specimens. To account for the diversity of DNA-positive samples, two different methods were applied to identify fungal species. First, direct sequencing allowed for the quick identification of pathogens that occur frequently in primarily mono-fungal specimens. Second, T-RFLP was used directly with isolated DNA of sample material to differentiate between mixed-*Candida* DNA without prior cultivation using a set of restriction enzymes that distinguishes up to 13 clinically relevant *Candida* species from a single sample at the same time. Therefore, the PCR/T-RFLP method presented here combines for the first time the detection of fungal species, the quantification of the most relevant *Candida* species, and the differentiation of mixed *Candida* strains without any use of conventional cultural techniques. This study evaluates the accuracy of this technique in detecting, quantifying and differentiating *Candida* species in colonized (polyfungal) and non-colonized body specimens. Furthermore, this study assesses the clinical relevance of *Candida* DNA in different body fluids from patients with liver cirrhosis.

## Results

### Patient characteristics

The following clinical and laboratory parameters were assessed at baseline (date of paracentesis and/or endoscopy): etiology of cirrhosis, sex, age, MELD score, Child-Pugh score, liver-function tests, white blood cell count (WBC), C-reactive protein (CrP), leukocyte count in ascitic fluid, and drug history. Patients were followed-up until October 2014 in order to capture fatalities and/or liver transplantations.

There were some significant differences between patients with and without cirrhosis at baseline. Parameters representing the liver function, such as bilirubin, albumin, Gamma-glutamyl-transferase (GGT), and INR, as well as the thrombocyte count, were significantly different in the cirrhosis group (Table 1). The C-reactive protein (CrP) level, which is a global marker for inflammation, was higher in individuals with cirrhosis, and the hemoglobin level was significantly better in patients without cirrhosis (Table 1). Patients with liver cirrhosis received more often an antibiotic treatment (21.5% vs 3.8%, p = 0.05) and had a higher number of endoscopic procedures (37.0% vs 19.2%, p<0.0001) in comparison to controls three months before duodenal fluid sampling. The use of proton pump inhibitors was not significantly different between both cohorts (Table 1).

**Table 1:** Patient characteristics at baseline.

Variable	Cirrhosis (n = 135)	Non-cirrhosis (n = 26)	Level of significance (p)
Age (years), median (range)	59 (25–87)	53 (22–88)	0.348
**Gender (male/female), n (%)**	**104/31 (77.0%/23.0%)**	**13/13 (50.0%/50.0%)**	**0.008**
Aetiology of cirrhosis, n (%) (n = 119)		Not applicable	
Alcoholic	97 (71.9)		
NASH	7 (5.2)		
Viral	8 (5.9)		
Cryptogenic	16 (11.9)		
Others	7 (5.2)		
Type of previous decompensation, n (%)			
(n = 135)		Not applicable	
Ascites	119 (88.1)		
Hepatic encephalopathy	46 (34.1)		
Bacterial infection	74 (54.8)		
Gastrointestinal hemorrhage	44 (32.6)		
Reason of hospital admission, n (%); (n = 135)		Not applicable	
Ascites	104 (77.0)		
Hepatic encephalopathy	25 (18.5)		
Bacterial infection	71 (52.6)		
Gastrointestinal hemorrhage	24 (17.8)		
**Previous endoscopies, n (%)**	**50 (37.0)**	**5 (19.2)**	**< 0.0001**
**MELD score, median (range)**	**14 (5–40)**	**Not applicable**	
**Bilirubin (μmol/l), median (range)**	**34.5 (3–643)**	**8.5 (6–25)**	**<0.0001**
**Albumin (g/l), median (range)**	**33.9 (16–47)**	**42.4 (32–49)**	**0.006**
**INR, median (range)**	**1.4 (1–3)**	**1 (1–1)**	**0.0001**
Serum creatinine (μmol/l), median (range)	90 (29–591)	72 (50–132)	0.059
GFR (ml/min), median (range)	72.8 (8–150)	87.6 (41–112)	0.313
ALAT (μkat/l), median (range)	0.4 (0–3)	0.34 (0–1)	0.787
**GGT (μkat/l); median (range)**	**1.6 (0–25)**	**0.64 (0–17)**	**0.04**
**Thrombocyte count (exp9/l), median (range)**	**124.5 (29–826)**	**233 (99–354)**	**<0.0001**
White blood cell count (exp9/l), median (range)	6.2 (0–33)	6.5 (2–15)	0.882
**Hemoglobin (mmol/l), median (range)**	**6.4 (4–10)**	**8.6 (5–10)**	**<0.0001**
**C-reactive protein (mg/dl), median (range)**	**21.7 (1–146)**	**1.8 (0–23)**	**<0.0001**
Antibiotic treatment, n (%)			
At Baseline	68 (50.4)	0 (0)	
3 months prior sampling	29 (21.5)	1 (3.8)	0.05
Proton pump inhibitors (n, %)	84 (62.2)	12 (46.2)	0.134

Categorical data are displayed as absolute and relative values and metric data as mean ± standard deviation or median (range), as appropriate. NASH = non-alcoholic steatohepatitis; GFR = glomerular filtration rate; INR = international normalized ratio; ALAT = Aspartat-Amino-Transferase; GGT = Gamma-glutamyltransferase.

### Detection and quantification of Candida DNA in biological specimens

**Fig 1:**
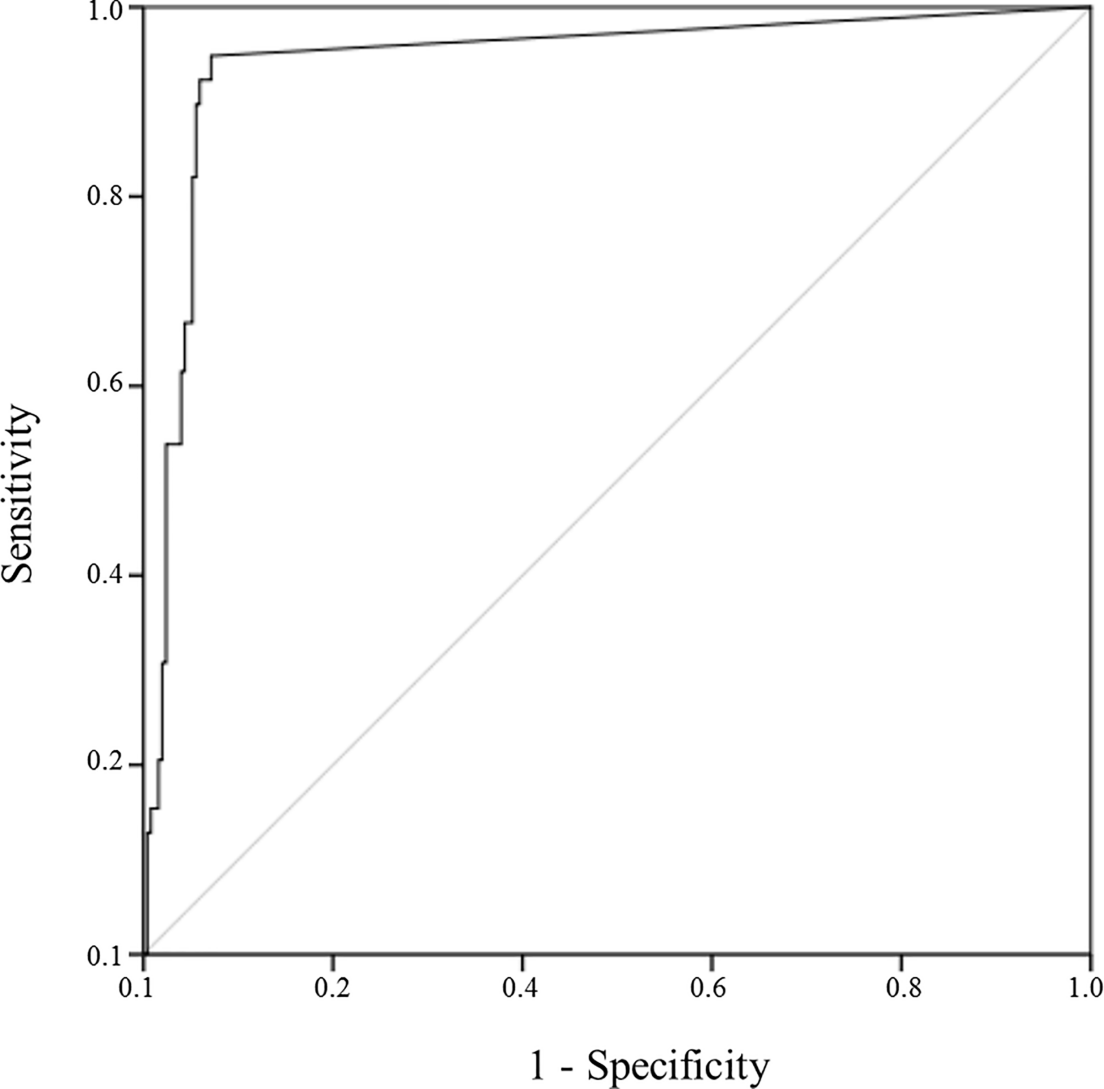
Receiver operating characteristic curve (ROC) of 18S rRNA gene based quantitative PCR from biological samples of patients with liver cirrhosis and controls.

We developed a real-time PCR for the detection and quantification of Candida DNA. DNase treatment was applied prior DNA isolation to ensure that the assay only captures DNA from intact fungal cells. Analytical sensitivity (limit of quantification) of the PCR was 370 copies ml^-1^ whereas clinical sensitivity was 96.1% compared to 71.0% using microbial culture as gold standard. Clinical specificity was 93.2% compared to 99.3% in culture methods. The predictive capacity to detect fungal pathogens proven by cultural techniques was calculated using the receiver operating characteristic curve (ROC curve, Fig 1). The area under the curve (AUROC) of 0.946 (95% CI 0.911–0.982; p<0.0001) and the Cohens kappa coefficient of κ = 0.779 (p<0.0001) confirmed the high concordance with conventional methods, and moreover, suggested the here described molecular methods as an effective and suitable tool for the detection and quantification of Candida DNA.

### Comparable fungal pathogen detection rates by using cultural and molecular methods

Microbial culture and PCR analyses in patients with cirrhosis showed low fungal detection rates in blood (culture: n = 1/135; 0.7%; PCR: n = 1/135; 0.7%) and ascites (culture: n = 0/92; 0%; n = 1/92; 1.1%). The level of *Candida* DNA was below 10^2^ copies/ml (blood: 3.0x10^1^; ascites: 1.3x10^1^) in both fungal DNA-positive samples (Table 2). In contrast, duodenal fluid of patients with cirrhosis was significantly colonized with fungal microorganisms. The culture-based detection rate of 66.7% was comparable to the PCR-based detection rate of 81.5% (p = 0.123). The PCR-based approach failed to detect fungal DNA in 5.6% (2 out of 36) of culture-positive duodenal fluids in cirrhosis but delivered additional positive results in 10 out of 18 (55.6%) culture-negative samples. In control patients, the frequency of fungal DNA detection (61.5% in controls vs. 81.5% in cirrhosis; p = 0.096) and culture positive results (46.2% in controls vs. 66.7% in cirrhosis; p = 0.093) in the duodenal samples was numerically but not statistically lower compared to patients with liver cirrhosis (Table 2). The median amount of *Candida* DNA in the duodenal fluid was 3.8x10^5^ 18S rRNA gene copies ml^-1^ in patients with cirrhosis (2.3x10^2^-6.3x10^9^) and therefore not different to 1.2x10^5^ 18S rRNA gene copies ml^-1^ in patients without cirrhosis (2.5x10^2^-2.4x10^7^, p = 0.618).

**Table 2:** Fungal pathogen and *Candida* DNA detection rates from microbial culture, real-time PCR analysis, and its statistical significance.

Patients with cirrhosis (n = 135)					Control patients without cirrhosis (n = 26)				
Sample material	No. of samples	Culture positive (%)	PCR positive (%), median copies/ml (range)	*P* value culture vs. PCR	No. of samples	Culture positive (%)	PCR positive (%), median copies/ml (range)	*P* value culture vs. PCR	*P* value PCR_cirrhosis_ vs. PCR_control_
Blood	n = 135	n = 1 (0.7%)	n = 1^a^ (0.7%), 3.0x10^1^		n = 26	n = 0 (0%)	n = 0 (0%)		
Ascites	n = 92	n = 0 (0%)	n = 1 (1.1%), 1.3x10^1^						
Duodenal fluid	n = 54	n = 36 (66.7%)	n = 44^b^ (81.5%), 3.8x10^5^	0.123	n = 26	n = 12 (46.2%)	n = 16^c^ (61.5%) 1.2x10^5^	0.404	0.096
			(2.3x10^2^-6.3x10^9^)				(2.5x10^2^-2.4x10^7^)		

^a^1/1

^b^34/36

^c^12/12 positive PCR results confirming microbial culture

### Antibacterial treatment enhances the duodenal Candida DNA level in patients with cirrhosis

Clinical factors that could potentially affect fungal DNA detection and *Candida* DNA quantification in cirrhosis were identified using a cross-sectional analysis at baseline. The PCR results for blood and ascites samples were not considered for this type of analysis because of their low detection rates.

At baseline, four out of 135 patients (3.0%) were treated with amphotericin B due to fungal esophagitis, and two patients (1.5%) were treated systemically with fluconazole due to suspected candidiasis. Moreover, 66 out of 135 patients (48.9%) were on antibiotic therapy, another 66 (48.9%) were receiving non-selective beta-blockers, and 82 (60.7%) were taking proton pump inhibitors. Antibacterial treatment did not affect the rate of fungal DNA detection in duodenal fluid but increased the amount of *Candida* DNA significantly by approximately two log levels (antibiotic treatment: 2.8x10^6^ [2.2x10^3^–4.4x10^8^] vs. no antibiotic treatment: 3.2 x 10^4^ [2.3x10^2^–6.3x10^9^]; p = 0.001). None of the other drugs were significantly associated with the presence or quantity of duodenal fungal DNA (S3 Table).

Neither gender, Child-Pugh score, MELD, etiology of liver disease, nor additional baseline parameters, as depicted in S1 and S2 Tables, were significantly associated with the presence or quantification of fungal DNA in duodenal fluid.

### Association of Candida DNA levels with survival of patients with cirrhosis

Although the survival curves of patients with and without Candida DNA diverged within 30 days after baseline showing a benefit for DNA negative patients, this result was statistically not significant (actuarial 30-day survival rate: DNA negative 100% vs. DNA positive 88.6%; p = 0.254; S1 Fig). The estimated overall survival of 517 days (95% CI 330–704) in patients with PCR-negative results was not different to 555 days (95% CI 447–662, p = 0.777) in patients with PCR-positive results. The survival rates were not associated by the cultural detection of fungal pathogens, whereas the *Candida* DNA quantification level was significantly associated with patients’ outcome. Increasing *Candida* DNA levels enhanced the risk for short-term death (30 day survival: HR 1.003 [1.001–1.005], p = 0.007.

### Fungal pathogen differentiation using cultural and molecular methods in non-colonized material

**Fig 2:**
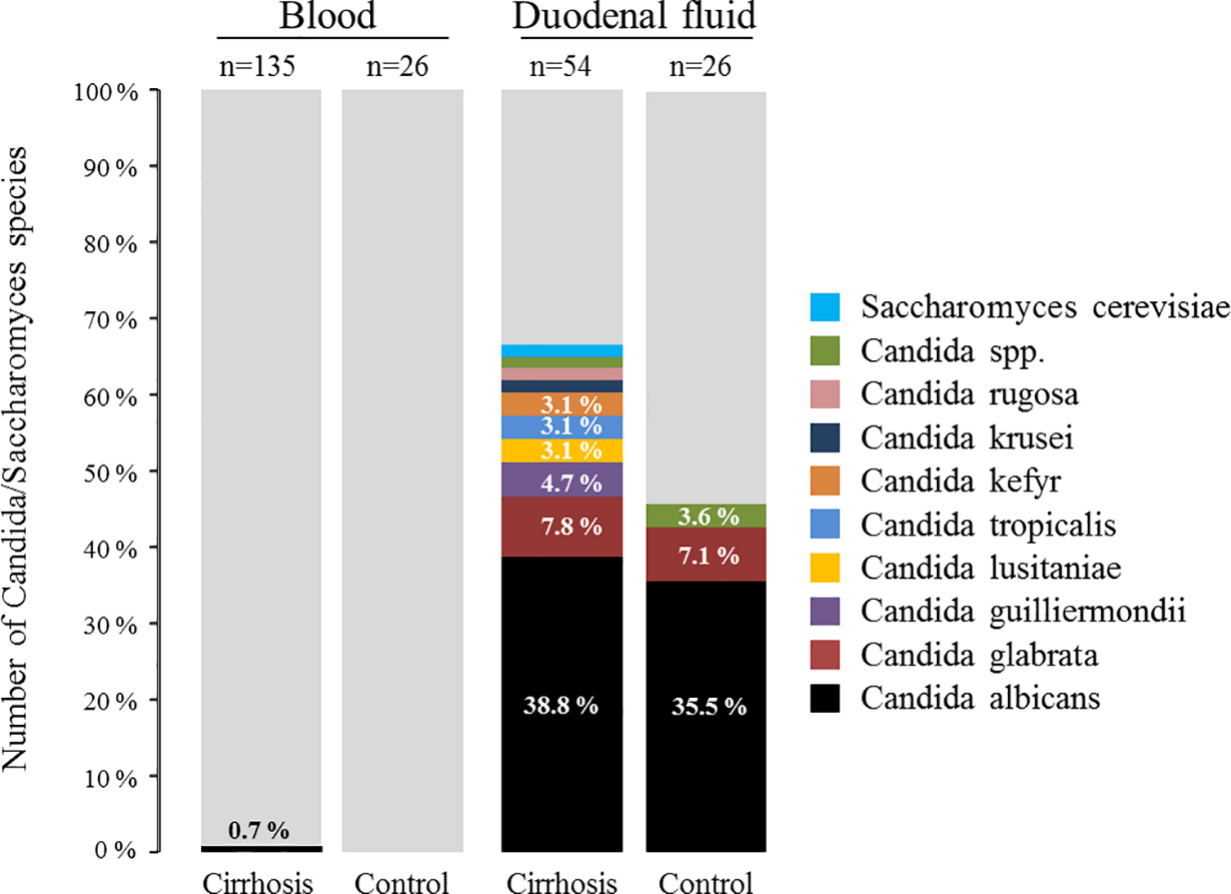
Detection of fungal species in blood and duodenal fluid of patients with liver cirrhosis and in control patients by using microbial culture techniques. The number of each sample group equals 100%. Stacked bars without percentage value correspond to 1.6%.

While no fungal pathogens were detected in the blood samples of the control patients, there was one positive blood result in a patient with cirrhosis that showed a monofungal infection with *Candida albicans* detected by microbial culture (Fig 2). This result was confirmed through direct sequencing of the PCR product. Although there were no culture-positive ascites samples, a PCR and subsequent sequence analysis identified *Saccharomyces cerevisiae* in one ascites sample (Table 2).

### Fungal pathogen differentiation using cultural and molecular methods in colonized material

*Candida albicans* was the predominant species detected by microbial culture methods in the duodenum of both patient groups, followed by *C*. *glabrata* (Fig 2). Less frequent *Candida* species, such as *C*. *guilliermondii*, *C*. *lusitaniae*, *C*. *tropicalis*, *C*. *kefyr*, *C*. *krusei* and *C*. *rugosa* were only captured in patients with liver cirrhosis. Non-*Candida* genera were not detected. Further microbial culture analyses revealed that seven out of 36 (19.4%) duodenal samples in cirrhosis and one out of 12 (8.3%) duodenal samples in controls (p = 0.659) were polyfungal, containing more than one *Candida* species.

**Fig 3:**
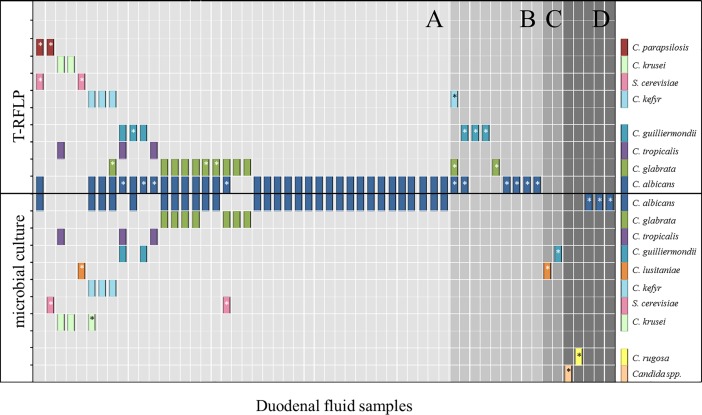
Fungal species analysis using either the T-RFLP or conventional culture methods on duodenal fluid samples from patients with liver cirrhosis and controls (n = 56). Each bar represents a single species. A star indicates that a fungal species could not be confirmed by the alternative method. The x-axis presents single duodenal samples so that multiple culture or T-RFLP-identified pathogens per sample are stacked in columns. Samples with culture-positive and PCR/T-RFLP-positive samples are compared in A (n = 40). Culture-negative but PCR/T-RFLP-positive samples are displayed in B (n = 9) and culture-positive and PCR/T-RFLP-negative samples are presented in C (n = 2). The duodenal samples that were culture-positive and PCR-positive but T-RFLP-negative are represented in area D (n = 5). Additional duodenal samples that were culture negative but PCR-positive with DNA not being sufficient for T-RFLP analysis (n = 3) have been left out in analysis.

Due to the high diversity in polyfungal samples, we established a T-RFLP analysis directly from isolated bacterial DNA to differentiate fungal pathogens to the species level and compared these data to results obtained through conventional cultural methods (Fig 3). In duodenal samples with positive fungal culture and valid PCR/T-RFLP results (n = 40), 92.3% (48/52) of *Candida* species (highlighted as bars in Fig 3) could be confirmed using T-RFLP. In these duodenal fluids, additional *Candida* species (n = 12, highlighted with stars) were detected in 27.5% samples (n = 11; Fig 3A).

The DNA quantity was adequate for the T-RFLP analysis in nine of 26 duodenal samples (34.6%) in which *Candida* DNA was identified, although the microbial culture results were negative (Fig 3B). These data contained sequences of *C*. *albicans*, *C*. *glabrata*, *C*. *guilliermondii*, and *C*. *kefyr*. The T-RFLP analysis could not be performed on seven out of 45 culture-positive samples (15.5%), as either the PCR was negative (two out of 45; Fig 3C) or the PCR product was quantitatively inadequate (five out of 45; Fig 3D).

## Discussion

Patients with end-stage liver diseases have an increased risk for infections with fungal pathogens resulting in devastating complications. Reliable diagnostic tests could facilitate early and adequate antifungal treatment, a potential lifesaving measure in this cohort [3, 18, 19]. Since cultural methods have a poor diagnostic quality and lack of providing reliable pathogen quantification levels, we established PCR-based techniques that allow us to detect panfungal 18S rRNA genes, and quantify and differentiate *Candida* DNA quickly.

These techniques were able to detect fungal pathogens accurately with a high concordance of about 96% compared to cultural techniques. Fungal DNA was amplified in additional 44% of culture-negative duodenal samples. This and the fact that overall fungal detection rate in duodenal fluid was higher with PCR (75%) than with conventional techniques (60%) clearly show that molecular tests can serve as an additional tool to capture fungal pathogens. However, it remains an open issue as to whether culture-negative but PCR-positive results have an actual clinical impact. The importance of PCR positive results is further questioned as we could not show an association between the mere fungal pathogen detection and patients’ clinical course. It is unlikely that the test captures contaminations leading to false-positive results, as blood and ascites samples were in almost all cases negative. Additionally, we have tested endoscopic channels regarding fungal impurities by analyzing the irrigation fluid after endoscope cleansing. Out of 24 samples only three were positive for fungal DNA and moreover had a very low quantification of 1.0x10^3^ copies ml^-1^. The fact that 97% of duodenal samples were above that quantification range speaks against a relevant test contamination.

However, it is potentially the patients’ selection which causes the lack of clinical association as the severity of the liver disease has been already identified as an important factor determining the risk for fungal infection [3]. Since patients with cirrhosis in this study had comparably low MELD scores of approximately 14, the risk for spontaneous invasive mycosis was presumably low and might explain the low fungal DNA detection rate in those primarily sterile body specimens. Recent studies have diagnosed *Candida*-specific bloodstream infections in 9% and 16% of patients already with moderately impaired liver function (MELD > 16) [18, 20]. Other fungal infections with *Aspergillus* or *Cryptococcus* species that were not detected in any of our samples have been found to be predominant in severely immunosuppressed patients, notably after liver transplantation [21, 22] or in patients with severe liver diseases [3] and a MELD score of 26. Therefore, performing a study in patients with higher risk for fungal infections, such as acute decompensated cirrhosis or acute-on-chronic liver failure, might be more appropriate to identify patients at risk for fungal infectious complications.

However, one significant strength of the here presented 18S rRNA gene-based PCR method is the ability to measure the *Candida* DNA quantitatively. The Candida DNA quantification level in the duodenal fluid was a potential predictor for patients’ short-term survival. The number of patients and events (n = 5) was inadequate to perform a multivariate Cox-regression analysis to adjust results to potential confounders, leaving the reliability of these results open so far. Besides, it is unclear whether fungal duodenal DNA directly leads to invasive infection and by that to fatalities in cirrhosis. At least, there is data suggesting that intestinal fungi might be the source for systemic spreading and infections via translocation [23]. The relevance of this mechanism is still not well explored in cirrhosis and the results shown here do not preclude that duodenal fungal DNA is just a surrogate marker for previous bacterial therapy. Therefore, further investigations are necessary to explore the mechanisms behind the association between fungal duodenal DNA and death. Yang and Schnabl have nicely shown that alcohol has been proven to induce a reduced intestinal fungal diversity, a Candida overgrowth and a translocation of fungal beta-glucan into the systemic circulation. Importantly, antifungal treatment decreased the fungal translocation and improved the liver damaging effect of alcohol [24]. Nevertheless, we did not find a significant association between alcoholic liver disease and presence or quantification of Candida DNA in duodenal fluid in our cohort (data not shown).

A pre-existing antibiotic treatment further increased the duodenal *Candida* DNA quantification level in cirrhosis. This fits to previous data showing an elevated fungal stool colonization in cancer patients after broad-spectrum antibiotic therapy [25, 26]. In this regard, Bajaj et al. reported, that patients with cirrhosis admitted due to bacterial infectious more likely developed fungal complications with a significant mortality rate of more than 50% [27]. As a consequence clinicians should closely review the indication for antibacterial treatments in patients with liver cirrhosis.

A further advantage of the PCR/T-RFLP method described here is the capacity to identify *Candida* species without the need for expertise in both handling and interpretation of fungal strains, which is necessary for culture techniques. PCR/T-RFLP overall confirmed 85% of strains identified by conventional methods and in 75% of culture-negative duodenal samples with positive PCR result additional species were identified. Thus, the application of this type of fungal pathogen identification and quantification is of high interest particularly in frequently colonized and polyfungal specimens where conventional methods certainly have a weakness.

It is important to mention that all analyses confirmed *Candida* species, in particular *Candida albicans*, as the predominant fungal pathogen. Van Thiel et al. [28] and Low and Rotstein [29] reported similar data, although non-albicans species, such as *C*. *glabrata*, *C*. *tropicalis*, and *C*. *parapsilosis*, obviously gain importance in our analysis.

The here presented method can have a weakness if the number of pathogens is low and only a small sample volume is screened. This could explain why the PCR failed to detect fungal DNA in two cases of culture-positive duodenal samples. Using larger volumes of sample material in DNA isolation and PCR analysis might overcome this problem.

Moreover, the fact that almost all blood and ascites samples were PCR negative might indicate that an intestinal translocation of fungal species as a source for blood stream infections might be overestimated. However, it should be considered that the maximum time span between ascites and duodenal sampling and blood sampling was five days. Since fungal pathogens probably occur temporarily, it might partially explain the low incidence of blood fungal DNA when measured in a single sample obtained from one time point.

In conclusion, the here presented PCR-based method reliably detects fungal DNA and is able to quantify and differentiate *Candida* species without any use of microbial culture methods. These methods are equally effective and quicker than conventional techniques and, therefore, could potentially serve as an additional diagnostic mean. Future prospective studies must address whether positive results in duodenal fluid could change the clinical management of patients with cirrhosis.

## Materials and methods

### Study design

This study included patients (n = 135) admitted with liver cirrhosis that were either decompensated with ascites or scheduled for an elective endoscopic procedure. The exclusion criteria included prior liver transplantation, malignancies other than hepatocellular carcinoma, and immunosuppressive therapy. Patients without liver diseases and infections who underwent endoscopy and did not receive antibiotic therapy at study enrolment served as the control group. Between May 2012 and March 2013, ascites samples (n = 92) and duodenal fluid samples (n = 73) were collected from 135 patients with cirrhosis and 26 non-liver disease patients at the University Hospital Leipzig. Corresponding blood samples were collected from each patient (n = 161) within five days. The study protocol conformed to the ethical guidelines of the 1975 Declaration of Helsinki and was approved by the ethics committee of the University of Leipzig (No. 356-10-13122010). All of the patients gave their written informed consent.

### Sample preparation

All samples were obtained during medically indicated procedure and were not part of the study protocol. In total, 3 ml blood and 50 ml ascites fluid were used for the analysis. In 30 out of 80 (37.5%) endoscopic procedures, an injection of 10 ml water was required to collect 3 ml of the duodenal fluid samples.

Blood, ascites, and duodenal fluids were analyzed using conventional microbiological culture methods to measure the presence of fungal pathogens. Ten milliliters of blood or ascites were inoculated in an aerobic and an anaerobic BacT/Alert blood culture bottle (bioMérieux, Marcy l’Étoile, France). A volume of 10μl of duodenal fluid was grown on Brucella-, Columbia blood-, and Columbia CAP selective agar, as well as on Endo-, Sabouraud-, and *Candida* ID agar. Additionally, 100 μl duodenal fluid or 300 μl in case samples were flushed were inoculated in Sabouraud bouillon to ensure fungal growth in samples with a low pathogen content. Isolated strains were identified using the Vitek 2 system (bioMérieux).

For the culture-independent analysis, 50 ml of ascites were centrifuged at 4500 rpm for 25 min in order to increase the number of fungal cells for DNA isolation and the pellet was resuspended in 3 ml supernatant. One ml of ascites pellet was used for DNA isolation which is comparable to 20 ml of ascites fluid for microbial cultural. Ascites pellets, fluid samples from duodenum, and whole-blood samples were stored with glycerin (final concentration of 20%) at -20°C until further sample processing.

### DNA-extraction

DNA was isolated from 1 ml resuspended ascites cell pellet, 1 ml whole blood, and 200 μl duodenal fluid using the MolYsis Complete5 (Molzym, Bremen, Germany) DNA isolation kit, as described previously [30], and subsequently analyzed using PCR. By using a DNase which is included in the DNA isolation kit we destroyed free circulating DNA and exclusively extracted DNA from intact fungal cells. The DNA isolation was performed in a HEPA-filtered hood with daily UV-radiation. A negative control using fetal calf serum instead of the sample material was included in each isolation series.

### Quantification of fungal DNA and sequence analysis

The Bioline SensiFast No Rox PCR Mastermix (Bioline, Luckenwalde, Germany) and the panfungal oligonucleotide primer pair 5’-ATT GGA GGG CAA GTC TGG TG-3’ and 5’-CCG ATC CCT AGT CGG CAT AG-3’ [11] covering the V4 variable region of the 18S rRNA gene were used to amplify the fungal DNA. In the same PCR approach, the hybridization probes, 5‘-LC Red640-CGA AAG TTA GGG GAT CGA AGA TG-3’ and 5’-CCA AGG ACG TTT TCA TTA ATC AAG A-Fl-3’, were used to quantify the *Candida* DNA [12] with a quantification limit of 370 copies ml^-1^ (experimentally measured and confirmed using probit analysis). The combination of using panfungal primers with *Candida*-specific probes in one PCR setting has two advantages. First, it enables the quantification of 18S rRNA gene copies of *Candida* species based on the determination of crossing points (Cp values) generated in quantitative PCR. Second, even if the sample does not contain *Candida*-derived DNA, non-*Candida* genera, such as *Aspergillus*, *Cryptococcus*, or *Fusarium*, can be qualitatively detected using the panfungal oligonucleotide pair and which are visible as a band after gel electrophoresis. Both amplicons, either from *Candida* or non-*Candida* genera, can be further analyzed using direct sequencing or T-RFLP. Each 20-μl-PCR reaction contained 10 μl of Mastermix, 1 μl of each primer (10 μM), 1 μl of each probe (5μM), 2.2 μl of PCR-grade water, and 5 μl of template DNA. Real-time PCR was performed on a LightCycler 480II instrument (Roche, Mannheim, Germany) with the following amplification steps: initial denaturation at 95°C for 10 min, 45 cycles of 15 sec at 95°C, 10 sec at 58°C, and 20 sec of 72°C with a fluorescence acquisition at the end of each 58°C step. A control using no template and a PCR-positive control containing *Candida albicans* DNA were included in each PCR run. All of the PCR reactions were subsequently subjected to agarose gel electrophoresis to obtain the correct sample size (approximately 500 bp) for each PCR product.

All PCR-positive blood and ascites samples with the appropriate size were purified and directly sequenced (GATC, Constance, Germany). Chromatograms were identified up to the genus level with a sequence identity higher than 98% using the BLAST tool [31].

For analysis of a possible contamination with Candida DNA from intact cells in the endoscopic working channel we analyzed the working and the auxiliary-water channel of three properly cleaned endoscopes. All samples were tested negative for fungal growth. Fungal DNA was isolated in quadruplicates from each sample and quantitative 18S rRNA gene based PCR revealed 3 out of 24 (12.5%) positive results for Candida DNA with a maximum of 1.0x10^3^ copies ml^-1^ deriving from *Candida guilliermondii* (using T-RFLP analysis).

### T-RFLP analysis for the differentiation of Candida DNA

For the selection of the restriction enzyme set in polyfungal samples, we used the most clinically relevant standard fungal strains obtained from the Institute for Laboratory Medicine, Clinical Chemistry and Molecular Diagnostics of University Hospital Leipzig: *C*. *albicans* (ATCC10231, ATCC14053, ZL13, ZL18, ZL23, ZL8b), *C*. *krusei* (ATCC6258, RV54, ZL11, ZL16, ZL20), *C*. *tropicalis* (ATCC13803, RV101, ZL9, ZL22), *C*. *glabrata* (ATCC2001, ZL12), and *C*. *parapsilosis* (ATCC22019), as well as sequenced strains from *C*. *haemulonii* (RV22), *C*. *dubliniensis* (RV30), *C*. *norvegensis* (RV31), *C*. *kefyr* (ZL17, ZL21), *C*. *guilliermondii* (ZL10), *C*. *sphaerica* (RV208), *C*. *lusitaniae* (ZL24), and *S*. *cerevisiae* (RV20, ZL14).

In order to show the phylogenetic relationship between these fungal strains, parsimony trees were calculated using the program package ARB [32] and added to the Silva small subunit ribosomal RNA reference database, version 108 [33] (S2 Fig).

The DNA from the standard strains, duodenal samples of patients with liver cirrhosis, and control patients that were PCR-positive, were amplified with the panfungal 18S rRNA-based oligonucleotide primer pair that was already used in the quantitative PCR, excluding an additional 5’-FAM-(6-carboxyfluorescein)-labeling of the forward primer. PCR was performed in a total volume of 25 μl containing 12.5 μl MyTaq HS Mix (Bioline), 1 μl of each primer (10 μM), 5.5 μl of PCR-grade water, and 5 μl of template DNA using a Veriti Thermal Cycler (Thermo Fisher Scientific, Waltham, USA). The amplification steps were as follows: initial denaturation at 95°C for 1 min, 40 cycles of 15 sec at 95°C, 15 sec at 55°C and 10 sec at 72°C, followed by a final elongation for 10 min at 72°C. The purification of PCR products was performed, as described above, and the DNA content of each PCR product was quantified using the Nanodrop ND-1000 spectrophotometer (Thermo Fisher Scientific).

After *in silico* enzyme digestion of all the standard strains using REPK Version 1.3 [34], a combination of five enzymes led to the best possible differentiation of the above pathogens: Hpy188I, BfaI, HaeIII, BglI and SmaI (New England Biolabs, Frankfurt, Germany).

For T-RFLP profiling, the PCR product from the standard strains (10 ng) and from the patient samples (40 ng) were separately digested for 3 h with 2 U of each restriction endonuclease at 37°C (Hpy188I, BfaI, HaeIII, BglI) or 25°C (SmaI). The cleaned DNA pellets of each sample were suspended in HiDi Formamide containing 1.5% MapMarker-1000 Rox standard (Applied Biosystems, Foster City, USA). The samples were denatured at 95°C for 10 min and subsequently chilled on ice. Using the ABI Prism 3100 genetic analyzer (Applied Biosystems), terminally labeled 18S rRNA gene fragments were separated using capillary electrophoresis. The lengths of the labeled terminal restriction fragments (T-RF) within the range of 50–510 bp were determined using Genemapper V3.7 software (Applied Biosystems). The *Candida* species were only identified successfully if the T-RF from all five enzymes showed the correct base-pair length (within the range of two base pairs), which corresponded to the reference strain presented in supplement material (S4 Table).

### Statistical analysis

Statistical analysis was performed by using SPSS 20 (SPSS, Illinois, USA). Categorical variables were displayed as percentages or frequencies, and the continuous variables were displayed as the mean ± standard deviation or the median and range, as appropriate. A two-sided p-value of < 0.05 was considered statistically significant. The comparison of unpaired samples was performed using a Mann-Whitney U test in the case of continuous data and a Chi-square test in the case of discrete data. For the correlation analysis, the Spearman-Rho coefficient was calculated and a correlation coefficient of r > 0.5 was considered relevant. A survival analysis was performed using Kaplan-Meier analysis, as appropriate, and compared using a Log-Rank test. Receiver-operator characteristic (ROC) curves were plotted and AUROC (area under the ROC curve) as well as κ coefficient were calculated.

## Supporting information

S1 Table Baseline parameters of patients with liver cirrhosis compared with *Candida* DNA-positive and -negative duodenal samples. (PDF)

S2 Table Correlation analysis using the Spearman Rho method between baseline parameters and Candida DNA quantification levels in duodenal samples. (PDF)

S3 Table Association between drug therapy and the presence and quantification of Candida DNA in duodenal fluid. (PDF)

S4 Table *In silico* and measured T-RF in reference to *Candida* strains. (PDF)

S1 Fig Thirty-day survival analysis using the Kaplan-Meier method. (TIF)

S2 Fig Parsimony tree depicting the relationship and affiliation between 18S rRNA gene sequences from *Candida* and *Saccharomyces* reference strains. The final position within the tree and bootstrap values were calculated using the ARB Parsimony Interactive tool (bootstrap values above 50% are shown; scale bar indicates 10% of estimated sequence divergence). The Genbank accession numbers for the reference strains were included and generated as part of this study.
(TIF)
