# Supplementary material for: Molecular quantification and differentiation of Candida species in biological specimens of patients with liver cirrhosis
Source: PLoS One. 2018 Jun 13;13(6):e0197319. doi: 10.1371/journal.pone.0197319 (PMC5999271; doi:10.1371/journal.pone.0197319)
Supplement: S1 Table — (PDF) [file pone.0197319.s001.pdf]

S1 Table. Baseline parameters of patients with liver cirrhosis compared with *Candida* DNA-positive and -negative duodenal samples.

Parameter	DNA-positive duodenal samples (n=44), median (range)	DNA-negative duodenal samples (n=10), median (range)	Level of significance (p)
MELD score, median (range)	14 (6-40)	12 (5-20)	0.242
Age (years), median (range)	61 (29-84)	57 (32–65)	0.063
Albumin (g/l), median (range)	35.4 (21–46)	31.3 (29–47)	0.675
Bilirubin (µmol/l), median (range)	36 (7–286)	35.6 (10–82)	0.672
INR, median (range)	1.3 (1–2)	1.35 (1–2)	0.927
Hemoglobin (mmol/l), median (range)	6.8 (5–9)	7 (5–8)	0.674
ALAT (µkat/l), median (range)	0.5 (0–3)	0.6 (0–2)	0.983
GGT (µkat/l), median (range)	1.6 (0–22)	1.6 (1-25)	0.749
Serum creatinine, (µmol/l), median (range)	87 (29–591)	67 (48–114)	0.243
GFR (ml/min), median (range)	80.3 (8-150)	91.5 (44-123)	0.364
White blood cell count (exp9/l), median (range)	5.75 (2–13)	5.4 (2–11)	0.779
C-reactive protein (mg/dl), median (range)	17.8 (1–104)	15.6 (1–44)	0.723

GFR = glomerular filtration rate; INR = international normalized ratio; ALAT = Aspartat-Amino-Transferase; GGT = Gamma-glutamyltransferase.
