# Supplementary material for: Molecular quantification and differentiation of Candida species in biological specimens of patients with liver cirrhosis
Source: PLoS One. 2018 Jun 13;13(6):e0197319. doi: 10.1371/journal.pone.0197319 (PMC5999271; doi:10.1371/journal.pone.0197319)
Supplement: S2 Table — (PDF) [file pone.0197319.s002.pdf]

S2 Table. Correlation analysis using the Spearman Rho method between baseline parameters and Candida DNA quantification levels in duodenal samples.

Parameter	Correlation coefficient (r)	Level of significance (p)
MELD score; median (range)	0.266	0.116
Age (years), median (range)	0.163	0.298
Albumin (g/l), median (range)	-0.258	0.286
Bilirubin (µmol/l), median (range)	0.028	0.867
INR, median (range)	0.073	0.773
Hemoglobin, (mmol/l), median (range)	-0.217	0.168
ALAT, (µkat/l), median (range)	-0.220	0.226
GGT, (µkat/l); median (range)	-0.216	0.234
Serum creatinine, (µmol/l), median (range)	0.162	0.304
GFR, (ml/min), median (range)	-0.142	0.368
White blood cell count (exp9/l), median (range)	-0.124	0.438
C-reactive protein (mg/dl), median (range)	0.209	0.229

GFR = glomerular filtration rate; INR = international normalized ratio; ALAT = Aspartat-Amino-Transferase; GGT = Gamma-glutamyltransferase.
