# Supplementary material for: Molecular quantification and differentiation of Candida species in biological specimens of patients with liver cirrhosis
Source: PLoS One. 2018 Jun 13;13(6):e0197319. doi: 10.1371/journal.pone.0197319 (PMC5999271; doi:10.1371/journal.pone.0197319)
Supplement: S3 Table — (PDF) [file pone.0197319.s003.pdf]

S3 Table. Association between drug therapy and the presence and quantification of Candida DNA in duodenal fluid.

Drug	Presence of fungal DNA (OR (IQR))	Level of significance (p)	Median DNA quantification (range)	Level of significance (p)
Antibiotic treatment (yes/no)	89.5% vs. 77.1% 2.5 (0.477–13.300)	0.277	**$2.8x10^{6}$ ($2.2x10^{3}$ –$4.4x10^{8}$) vs. $3.2x10^{4}$ ($2.3x10^{2}$–$6.3x10^{9}$)**	**0.001**
Betablocker (yes/no)	80.0% vs. 84.2% 0.704 (0.302–5.889)	0.737	$2.5x10^{5}$ ($2.3x10^{2}$–$6.3x10^{9}$) vs. $1.3x10^{6}$ ($8.8x10^{2}$–$4.1x10^{9}$)	0.390
Protone pump inhibitors (yes/no)	89.3% vs. 73.1% 0.137 (0.74–1.428)	0.169	$5.0x10^{5}$ ($2.3x10^{2}$–$4.1x10^{9}$) vs. $1.2x10^{5}$ ($7.8x10^{2}$–$6.3x10^{9}$)	0.818

OR = odds ratio; IQR = interquartile range
