# Supplementary material for: Molecular quantification and differentiation of Candida species in biological specimens of patients with liver cirrhosis
Source: PLoS One. 2018 Jun 13;13(6):e0197319. doi: 10.1371/journal.pone.0197319 (PMC5999271; doi:10.1371/journal.pone.0197319)
Supplement: S4 Table — (PDF) [file pone.0197319.s004.pdf]

S4 Table. *In silico* and measured T-RF in reference to *Candida* strains.

Strains	Measured (in silico) T-RF [bp]				
	**BfaI**	**BglI**	**HaeIII**	**Hpy188I**	**SmaI**
***C. albicans* ATCC10231[a)]**	285.12 (285)	494.39 (490)	104.53 (108)	342.23 (341)	494.37 (490)
***C. tropicalis* ATCC13803[b)]**	159.29 (160)	492.91 (488)	105.00 (108)	120.03 (120)	493.80 (488)
***C. kefyr* ZL17[c)]**	300.10 (298)	508.68 (503)	105.52 (108)	112.17 (115)	509.24 (503)
***C. glabrata* ATCC2001[d)]**	300.29 (298)	101.89 (105)	95.47 (99)	111.41 (114)	139.77 (142)
***C. haemulonii* RV22**	278.24 (283)	488.03 (488)	488.09 (488)	335.25 (339)	487.83 (488)
***C. lusitaniae* ZL24**	272.55 (276)	482.35 (481)	482.51 (481)	329.31 (332)	482.41 (481)
***C. parapsilosis* ATCC22019**	160.17 (160)	169.52 (170)	104.68 (108)	344.00 (341)	494.82 (490)
***C. dubliniensis* RV30**	158.87 (160)	495.08 (490)	104.48 (108)	343.09 (341)	495.34 (490)
***C. guilliermondii* ZL10**	299.42 (298)	119.48 (122)	105.38 (108)	356.49 (354)	509.54 (503)
***C. sphaerica* RV208**	299.35 (298)	508.41 (503)	508.68 (503)	112.38 (115)	508.75 (503)
***C. krusei* ATCC6258[e)]**	156.23 (157)	488.96 (484)	95.49 (99)	224.78 (224)	488.56 (484)
***C. norvegensis* RV31**	155.57 (157)	496.77 (492)	95.49 (99)	344.98 (343)	97.44 (102)

100% homology for following strains: *a) C. albicans* ATCC10231 and ATCC14053, ZL8b, ZL13, ZL18, ZL23*; b) C. tropicalis* ATCC13803 and RV101, ZL9, ZL22*; c) C. kefyr* ZL17 and ZL21*; d) C. glabrata* ATCC2001 and ZL12*; e) C. krusei* ATCC6258 and RV54, ZL11, ZL16, ZL20*; f) S. cerevisiae* RV20 and ZL14.
