# Supplementary figures and images for: Molecular quantification and differentiation of Candida species in biological specimens of patients with liver cirrhosis
Source: PLoS One. 2018 Jun 13;13(6):e0197319. doi: 10.1371/journal.pone.0197319 (PMC5999271; doi:10.1371/journal.pone.0197319)

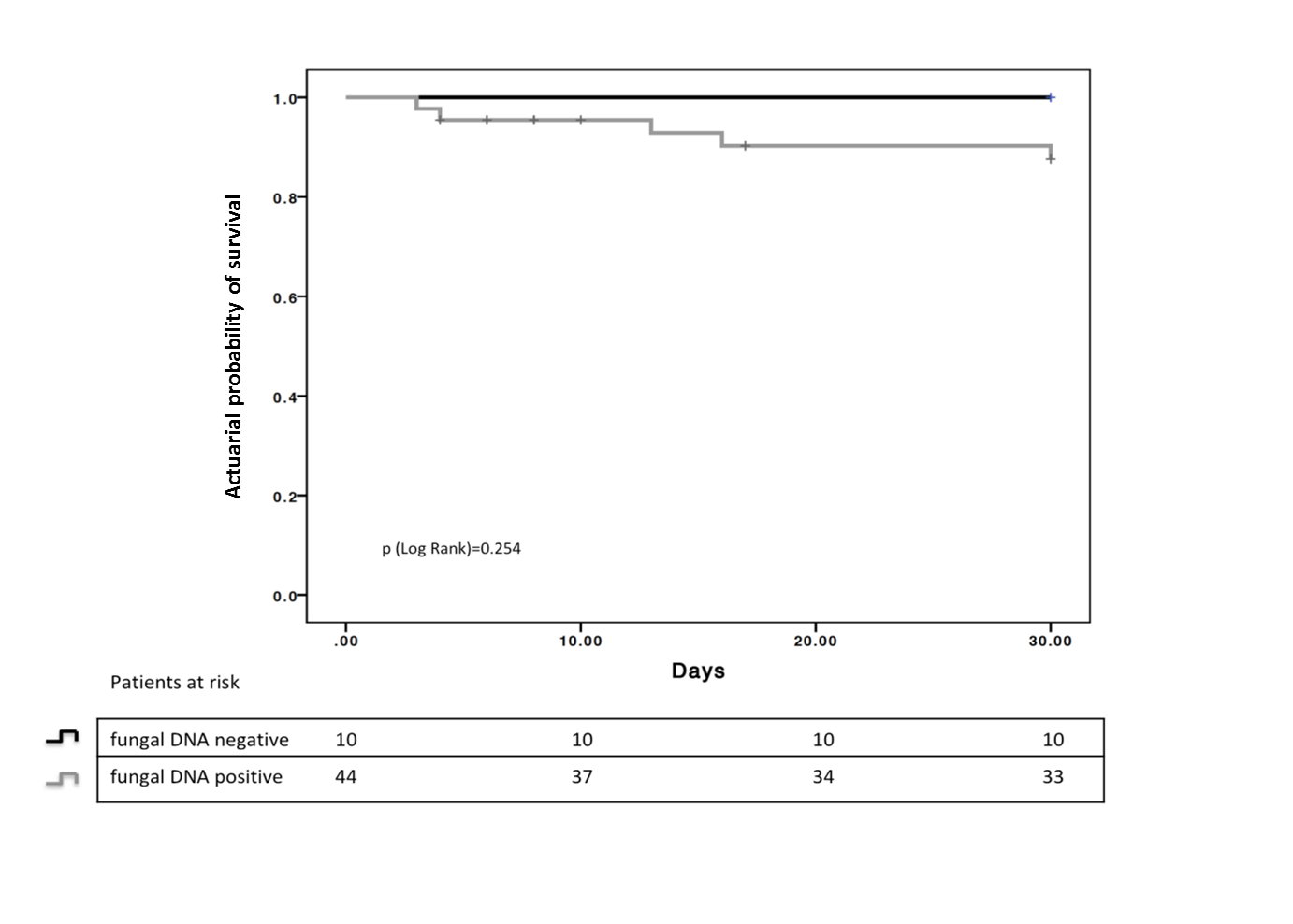

Supplement: S1 Fig — (TIF) [file pone.0197319.s005.tif]

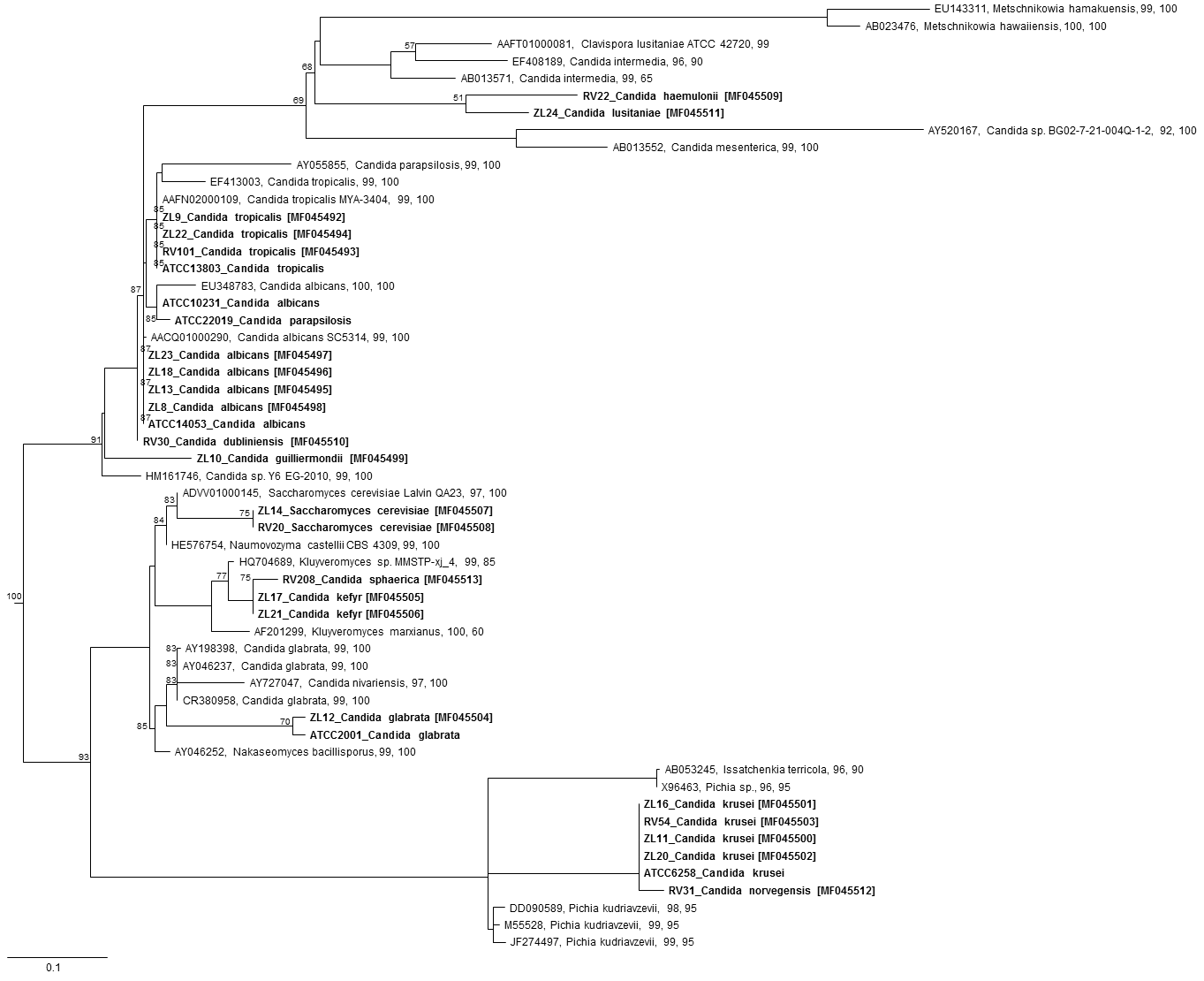

Supplement: S2 Fig — The final position within the tree and bootstrap values were calculated using the ARB Parsimony Interactive tool (bootstrap values above 50% are shown; scale bar indicates 10% of estimated sequence divergence). The Genbank accession numbers for the reference strains were included and generated as part of this study. (TIF) [file pone.0197319.s006.tif]
